# 3-[2-(2,6-Dichloro­anilino)benz­yl]-4-[(4-meth­oxy­benzyl­idene)amino]-1*H*-1,2,4-triazole-5(4*H*)-thione

**DOI:** 10.1107/S1600536811034799

**Published:** 2011-08-31

**Authors:** M. Vinduvahini, K. R. Roopashree, Suman Bhattacharya, K. Mohan Krishna, Venkatesh B. Devaru

**Affiliations:** a Department of Physics, Sri D Devaraja Urs Govt. First Grade College, Hunsur 571 105, Mysore District, Karnataka, India; b Department of Physics, Yuvaraja’s College (Constituent College), University of Mysore, Mysore 570 005, Karnataka, India; c Department of Chemistry, Pondicherry University, Pondicherry 605 014, India; d Department of Pharmacy, JSS College of Pharmacy, Mysore 570015, Karnataka, India; e Department of P.G. Studies in Physics, L V D College, Raichur 584 103, Karnataka, India.

## Abstract

In the title compound, C_23_H_19_Cl_2_N_5_OS, the triazole ring makes dihedral angles of 24.81 (18), 69.94 (19) and 35.68 (18)° with the dichloro­phenyl, benzene and meth­oxy­phenyl rings, respectively. An intra­molecular N—H⋯N hydrogen bond occurs. In the crystal, inversion dimers linked by pairs of N—H⋯S hydrogen bonds occur. In addition, there are weak C—H⋯π inter­actions involving the dichloro­phenyl and triazole rings.

## Related literature

For general background to Schiff bases, see: Dhar & Taploo (1982 ▶). For the biological and pharmaceutical activity of related compounds, see: Kiran *et al.* (2006 ▶); Shi *et al.* (2007 ▶); Dharmarajan *et al.* (2006 ▶); Hearn & Cynamon (2004 ▶); Dimova *et al.* (2001 ▶). For a related structure, see: Yang *et al.* (2005 ▶).
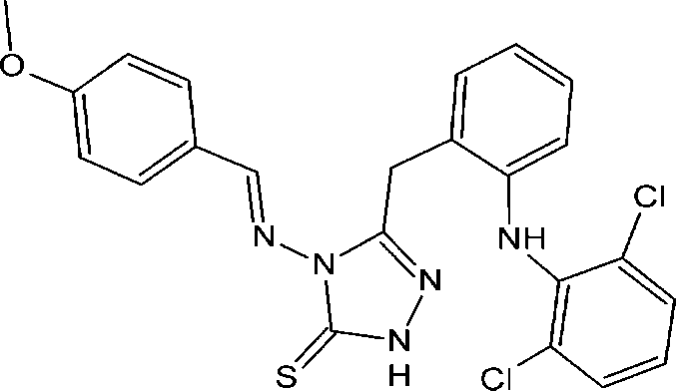


## Experimental

### 

#### Crystal data

C_23_H_19_Cl_2_N_5_OS
*M* *_r_* = 484.39
Triclinic, 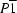

*a* = 7.9438 (4) Å
*b* = 10.9163 (7) Å
*c* = 14.0384 (8) Å
α = 75.332 (5)°
β = 75.807 (5)°
γ = 88.410 (5)°
*V* = 1140.98 (11) Å^3^
*Z* = 2
Mo *K*α radiation
μ = 0.40 mm^−1^
*T* = 293 K
0.22 × 0.15 × 0.12 mm

#### Data collection

Oxford Diffraction Xcalibur diffractometer
Absorption correction: multi-scan (*CrysAlis PRO RED*; Oxford Diffraction, 2010 ▶) *T*_min_ = 0.790, *T*_max_ = 1.000
7560 measured reflections
4009 independent reflections
2735 reflections with *I* > 2σ(*I*)
*R*_int_ = 0.025

#### Refinement

*R*[*F* ^2^ > 2σ(*F* ^2^)] = 0.057
*wR*(*F* ^2^) = 0.159
*S* = 1.06
4009 reflections
289 parameters
H-atom parameters constrained
Δρ_max_ = 0.37 e Å^−3^
Δρ_min_ = −0.44 e Å^−3^

### 

Data collection: *CrysAlis PRO CCD* (Oxford Diffraction, 2010 ▶); cell refinement: *CrysAlis PRO CCD*; data reduction: *CrysAlis PRO RED* (Oxford Diffraction, 2010 ▶); program(s) used to solve structure: *SHELXS97* (Sheldrick, 2008 ▶); program(s) used to refine structure: *SHELXL97* (Sheldrick, 2008 ▶); molecular graphics: *ORTEP-3* (Farrugia, 1997 ▶) and *CAMERON* (Watkin *et al.*, 1993 ▶); software used to prepare material for publication: *WinGX* (Farrugia, 1999 ▶).

## Supplementary Material

Crystal structure: contains datablock(s) I, global. DOI: 10.1107/S1600536811034799/wn2450sup1.cif

Structure factors: contains datablock(s) I. DOI: 10.1107/S1600536811034799/wn2450Isup2.hkl

Supplementary material file. DOI: 10.1107/S1600536811034799/wn2450Isup3.cml

Additional supplementary materials:  crystallographic information; 3D view; checkCIF report

## Figures and Tables

**Table 1:** Hydrogen-bond geometry (Å, °) *Cg*1 is the centroid of the triazole ring.

*D*—H⋯*A*	*D*—H	H⋯*A*	*D*⋯*A*	*D*—H⋯*A*
N5—H5⋯N6	0.86	2.35	3.047 (4)	139
N8—H8⋯S3^i^	0.86	2.40	3.246 (3)	170
C11—H11⋯*Cg*1^ii^	0.93	2.79	3.465 (4)	125

Symmetry codes: (i) 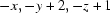; (ii) 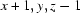.

**Fig. 1.**
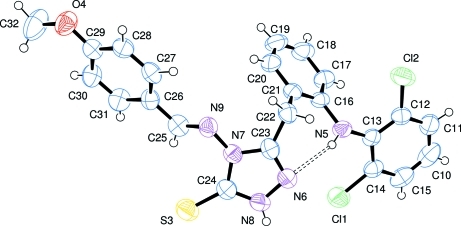
The title molecule with the displacement ellipsoids drawn at the 50% probability level. The H atoms are shown as spheres of arbitrary radii. The dashed line indicates the intramolecular hydrogen bond.

**Fig. 2.**
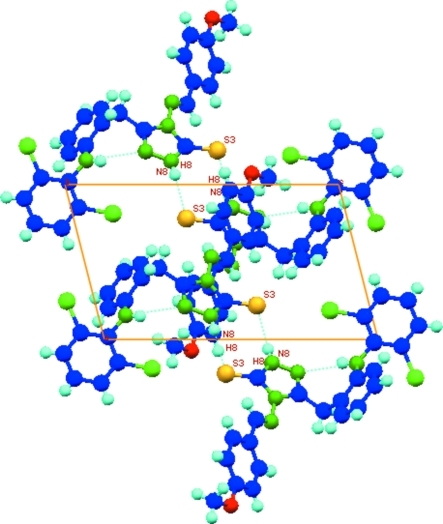
The crystal structure viewed down the b axis.

## supplementary crystallographic information

### Comment

Schiff bases are condensation products of primary amines with carbonyl compounds. The presence of the lone pair of electrons in the sp^2^ hybridized orbital of the nitrogen atom of the azomethine group is of considerable chemical and biological importance. Schiff bases are some of the most widely used organic compounds. They are used as pigments and dyes, catalysts, intermediates in organic synthesis, and polymer stabilisers (Dhar & Taploo, 1982). They have also been shown to exhibit a broad range of biological properties, including antimalarial, antibacterial, antifungal, antiviral and antitubercular activities (Kiran *et al.*, 2006; Shi *et al.*, 2007; Dharmarajan *et al.*, 2006; Hearn & Cynamon, 2004). Imine or azomethine groups are present in various natural, natural-derived and non-natural compounds. The imine group present in such compounds has been shown to be critical to their biological activities (Dimova *et al.*, 2001).

The asymmetric unit of  5-[2-[(2,6-dichlorophenyl)amino]benzyl]-4- (4-methoxybenzylideneamino)-2*H*-1,2,4-triazole-3(4*H*)-thione, C_23_H_19_Cl_2_N_5_OS, contains one molecule (Fig. 1). The triazole ring makes dihedral angles of 24.81 (18)°, 69.94 (19)° and 35.68 (18)° with the dichlorophenyl, benzene and methoxyphenyl rings (C10–C15), (C16–C21) and (C26–C31), respectively. The bond distances and angles are in good agreement with those in a related crystal structure (Yang *et al.*, 2005). In the crystal, the structure is stabilized by intramolecular N5—H5···N6 and intermolecular N8—H8···S3 hydrogen bonds (Table 1). In addition, there are weak C—H···π interactions involving the dichlorophenyl and triazole rings. In the crystal structure, molecules are stacked along the *b* axis (Fig. 2).

### Experimental

An equimolar mixture of thiocarbohydrazide (TCH) and diclofeac was mixed and heated gently on an oil bath until the evolution of H_2_S ceased. The reaction mixture was then cooled to room temperature and poured into ice cold water and stirred well. The resulting product was filtered, dried and recrystallized to obtain 3-[2-[(2,6-dichlorophenyl) amino] benzyl]-4-amino-5-mercapto(4*H*)-1,2,4-triazole.

To a solution of 3-[2-[(2,6-dichlorophenyl) amino] benzyl]-4-amino-5-mercapto(4*H*)- 1,2,4-triazole (10 mmol) in glacial acetic acid (15 ml) was added 10 mmol of anisaldehyde. The reaction mixture was then refluxed for 4 h. The precipitated solid obtained after the elimination of glacial acetic acid was washed with cold water and filtered. The solid obtained was then recrystallized using methanol (yield-86%). *M*.p. 507–509 K.

### Refinement

All H atoms were placed at calculated positions and refined using a riding model. N—H = 0.86 Å, C—H = 0.97 Å for methylene, C—H = 0.93 Å for aromatic and C—H = 0.96 Å for methyl. *U_iso_(H)* = 1.5*U_eq_(C)* for methyl H and 1.2*U_eq_(C, N)* for all other H atoms.

### Crystal data

**Table:**

C_23_H_19_Cl_2_N_5_OS	*Z* = 2
*M**_r_* = 484.39	*F*(000) = 500
Triclinic, *P*1	*D*_x_ = 1.410 Mg m^−^^3^
Hall symbol: -P 1	Melting point: 509 K
*a* = 7.9438 (4) Å	Mo *K*α radiation, λ = 0.71073 Å
*b* = 10.9163 (7) Å	Cell parameters from 4009 reflections
*c* = 14.0384 (8) Å	θ = 2.7–25.0°
α = 75.332 (5)°	µ = 0.40 mm^−^^1^
β = 75.807 (5)°	*T* = 293 K
γ = 88.410 (5)°	Prism, colourless
*V* = 1140.98 (11) Å^3^	0.22 × 0.15 × 0.12 mm

### Data collection

**Table:**

Oxford Diffraction Xcalibur diffractometer	4009 independent reflections
Radiation source: fine-focus sealed tube	2735 reflections with *I* > 2σ(*I*)
graphite	*R*_int_ = 0.025
Detector resolution: 15.9821 pixels mm^-1^	θ_max_ = 25.0°, θ_min_ = 2.7°
ω scans	*h* = −9→9
Absorption correction: multi-scan (*CrysAlis PRO RED*; Oxford Diffraction, 2010)	*k* = −12→12
*T*_min_ = 0.790, *T*_max_ = 1.000	*l* = −16→16
7560 measured reflections	

### Refinement

**Table:**

Refinement on *F*^2^	Primary atom site location: structure-invariant direct methods
Least-squares matrix: full	Secondary atom site location: difference Fourier map
*R*[*F*^2^ > 2σ(*F*^2^)] = 0.057	Hydrogen site location: inferred from neighbouring sites
*wR*(*F*^2^) = 0.159	H-atom parameters constrained
*S* = 1.06	*w* = 1/[σ^2^(*F*_o_^2^) + (0.0714*P*)^2^ + 0.2386*P*] where *P* = (*F*_o_^2^ + 2*F*_c_^2^)/3
4009 reflections	(Δ/σ)_max_ < 0.001
289 parameters	Δρ_max_ = 0.37 e Å^−^^3^
0 restraints	Δρ_min_ = −0.44 e Å^−^^3^

### Special details

**Table:**

Experimental. *CrysAlis PRO*, Oxford Diffraction Ltd., Version 1.171.33.55 (release 05–01–2010 CrysAlis171. NET) Empirical absorption correction using spherical harmonics, implemented in SCALE3 ABSPACK scaling algorithm.Elemental analysis for C_23_H_19_C_l2_N_5_OS (484): Calculated C 57.03, H 3.95, N 14.46; Found C 56.65, H 3.87, N 14.39. IR (ν cm^-1^, KBr): 3331 (NH), 2931 (C—H aliphatic), 1604 (C═N imine linkage), 1257 (C═S), 1153 (C—O of methoxy group). 1H NMR (DMSO):δ (p.p.m.) = 3.80 (s, 3H, 4- OCH_3_), 4.23 (s, 2H, Ar—CH_2_), 6.19 (s, 1H, Ar—NH), 7.27–6.97 (m, 5H, Ar—H), 7.35 (d, 2H, Ar—H), 7.48 (d, 2H, Ar—H), 7.64 (d, 2H, Ar—H), 9.85 (s, 1H, CH), 13.46 (s, 1H, NH). 13 C NMR:δ (p.p.m.) = 28.05 (Ar—CH_2_), 121–134 (aromatic carbons), 149.52 (C_5_-of 1,2,4-triazole), 161.65 (C of imine linkage), 163.17 (C_3_-of 1,2,4-triazole).
Geometry. All s.u.'s (except the s.u. in the dihedral angle between two l.s. planes) are estimated using the full covariance matrix. The cell s.u.'s are taken into account individually in the estimation of s.u.'s in distances, angles and torsion angles; correlations between s.u.'s in cell parameters are only used when they are defined by crystal symmetry. An approximate (isotropic) treatment of cell s.u.'s is used for estimating s.u.'s involving l.s. planes.
Refinement. Refinement of *F*^2^ against ALL reflections. The weighted *R*-factor *wR* and goodness of fit *S* are based on *F*^2^, conventional *R*-factors *R* are based on *F*, with *F* set to zero for negative *F*^2^. The threshold expression of *F*^2^ > 2σ(*F*^2^) is used only for calculating *R*-factors(gt) *etc*. and is not relevant to the choice of reflections for refinement. *R*-factors based on *F*^2^ are statistically about twice as large as those based on *F*, and *R*- factors based on ALL data will be even larger.

### Fractional atomic coordinates and isotropic or equivalent isotropic displacement parameters (Å^2^)

**Table:**

	*x*	*y*	*z*	*U*_iso_*/*U*_eq_	
Cl1	−0.18788 (15)	0.88750 (10)	0.85215 (8)	0.0827 (4)	
Cl2	0.24757 (15)	0.78540 (10)	1.10115 (8)	0.0812 (4)	
S3	0.22012 (14)	0.90073 (10)	0.41182 (7)	0.0742 (3)	
O4	1.0724 (3)	0.4658 (2)	0.3076 (2)	0.0755 (7)	
N5	0.1553 (4)	0.8259 (2)	0.89938 (19)	0.0510 (7)	
H5	0.1832	0.8940	0.8513	0.061*	
N6	0.2131 (4)	0.9612 (3)	0.6750 (2)	0.0594 (8)	
N7	0.3781 (3)	0.8614 (2)	0.57024 (18)	0.0495 (7)	
N8	0.1506 (4)	0.9675 (3)	0.5909 (2)	0.0597 (8)	
H8	0.0572	1.0054	0.5821	0.072*	
N9	0.5297 (4)	0.8041 (3)	0.53284 (19)	0.0546 (7)	
C10	−0.2520 (6)	0.8508 (3)	1.1467 (3)	0.0814 (14)	
H10	−0.3429	0.8562	1.2015	0.098*	
C11	−0.0915 (6)	0.8223 (3)	1.1616 (3)	0.0696 (11)	
H11	−0.0726	0.8084	1.2262	0.083*	
C12	0.0435 (5)	0.8140 (3)	1.0804 (3)	0.0561 (9)	
C13	0.0187 (4)	0.8306 (3)	0.9827 (2)	0.0461 (8)	
C14	−0.1480 (5)	0.8606 (3)	0.9718 (3)	0.0543 (9)	
C15	−0.2817 (5)	0.8718 (3)	1.0521 (3)	0.0702 (11)	
H15	−0.3913	0.8934	1.0422	0.084*	
C16	0.2500 (4)	0.7173 (3)	0.8888 (2)	0.0464 (8)	
C17	0.2012 (5)	0.6003 (3)	0.9570 (3)	0.0574 (9)	
H17	0.1039	0.5925	1.0111	0.069*	
C18	0.2970 (6)	0.4949 (3)	0.9447 (3)	0.0718 (11)	
H18	0.2648	0.4171	0.9916	0.086*	
C19	0.4378 (6)	0.5039 (4)	0.8646 (4)	0.0759 (12)	
H19	0.5003	0.4326	0.8560	0.091*	
C20	0.4865 (5)	0.6194 (4)	0.7968 (3)	0.0650 (10)	
H20	0.5825	0.6250	0.7423	0.078*	
C21	0.3969 (4)	0.7281 (3)	0.8072 (2)	0.0494 (8)	
C22	0.4599 (4)	0.8547 (3)	0.7349 (2)	0.0547 (8)	
H22A	0.4575	0.9172	0.7735	0.066*	
H22B	0.5793	0.8488	0.6985	0.066*	
C23	0.3522 (5)	0.8972 (3)	0.6606 (2)	0.0521 (8)	
C24	0.2465 (5)	0.9101 (3)	0.5241 (2)	0.0544 (9)	
C25	0.5168 (5)	0.7271 (3)	0.4798 (2)	0.0581 (9)	
H25	0.4086	0.7128	0.4694	0.070*	
C26	0.6628 (5)	0.6615 (3)	0.4353 (2)	0.0518 (8)	
C27	0.8297 (5)	0.6764 (3)	0.4456 (2)	0.0558 (9)	
H27	0.8513	0.7316	0.4823	0.067*	
C28	0.9630 (5)	0.6107 (3)	0.4021 (2)	0.0573 (9)	
H28	1.0745	0.6220	0.4091	0.069*	
C29	0.9330 (5)	0.5268 (3)	0.3474 (2)	0.0541 (8)	
C30	0.7691 (5)	0.5119 (3)	0.3362 (3)	0.0592 (9)	
H30	0.7479	0.4564	0.2996	0.071*	
C31	0.6365 (5)	0.5787 (3)	0.3789 (3)	0.0640 (10)	
H31	0.5259	0.5687	0.3700	0.077*	
C32	1.0423 (6)	0.3783 (5)	0.2520 (4)	0.1100 (17)	
H32A	1.1497	0.3409	0.2272	0.165*	
H32B	0.9973	0.4224	0.1956	0.165*	
H32C	0.9599	0.3130	0.2959	0.165*	

### Atomic displacement parameters (Å^2^)

**Table:**

	*U*^11^	*U*^22^	*U*^33^	*U*^12^	*U*^13^	*U*^23^
Cl1	0.0819 (8)	0.0906 (7)	0.1017 (8)	0.0203 (6)	−0.0537 (6)	−0.0428 (6)
Cl2	0.0908 (8)	0.0765 (7)	0.0882 (7)	0.0164 (6)	−0.0510 (6)	−0.0159 (5)
S3	0.0749 (7)	0.0830 (7)	0.0687 (6)	0.0060 (6)	−0.0279 (5)	−0.0169 (5)
O4	0.0627 (17)	0.0854 (18)	0.0940 (18)	0.0095 (14)	−0.0230 (14)	−0.0482 (15)
N5	0.0553 (18)	0.0385 (14)	0.0511 (15)	0.0053 (12)	−0.0086 (13)	−0.0019 (12)
N6	0.061 (2)	0.0668 (18)	0.0522 (16)	0.0066 (16)	−0.0165 (14)	−0.0156 (14)
N7	0.0478 (17)	0.0534 (16)	0.0455 (14)	−0.0031 (13)	−0.0115 (13)	−0.0089 (12)
N8	0.0598 (19)	0.0663 (18)	0.0539 (16)	0.0106 (15)	−0.0212 (15)	−0.0108 (14)
N9	0.0556 (19)	0.0581 (17)	0.0484 (15)	0.0009 (14)	−0.0095 (13)	−0.0134 (13)
C10	0.088 (3)	0.052 (2)	0.075 (3)	0.006 (2)	0.022 (2)	−0.004 (2)
C11	0.100 (3)	0.049 (2)	0.049 (2)	0.010 (2)	−0.009 (2)	−0.0045 (16)
C12	0.070 (2)	0.0380 (17)	0.057 (2)	0.0060 (16)	−0.0170 (18)	−0.0043 (15)
C13	0.054 (2)	0.0304 (15)	0.0510 (18)	0.0007 (14)	−0.0115 (16)	−0.0068 (14)
C14	0.057 (2)	0.0383 (17)	0.071 (2)	0.0026 (15)	−0.0199 (19)	−0.0160 (16)
C15	0.052 (2)	0.051 (2)	0.098 (3)	0.0028 (17)	−0.004 (2)	−0.015 (2)
C16	0.0443 (19)	0.0424 (17)	0.0592 (19)	0.0039 (15)	−0.0234 (16)	−0.0147 (15)
C17	0.060 (2)	0.0435 (19)	0.068 (2)	−0.0002 (17)	−0.0195 (18)	−0.0086 (17)
C18	0.088 (3)	0.045 (2)	0.092 (3)	0.011 (2)	−0.043 (3)	−0.017 (2)
C19	0.081 (3)	0.063 (3)	0.104 (3)	0.035 (2)	−0.049 (3)	−0.036 (2)
C20	0.049 (2)	0.083 (3)	0.078 (2)	0.022 (2)	−0.0300 (19)	−0.038 (2)
C21	0.0428 (19)	0.059 (2)	0.0560 (19)	0.0041 (16)	−0.0241 (16)	−0.0199 (16)
C22	0.046 (2)	0.071 (2)	0.0493 (18)	−0.0040 (17)	−0.0130 (15)	−0.0165 (17)
C23	0.055 (2)	0.0539 (19)	0.0455 (18)	−0.0076 (17)	−0.0093 (16)	−0.0105 (15)
C24	0.056 (2)	0.0509 (19)	0.0497 (19)	−0.0072 (17)	−0.0087 (17)	−0.0047 (16)
C25	0.055 (2)	0.057 (2)	0.059 (2)	−0.0110 (17)	−0.0118 (17)	−0.0092 (18)
C26	0.055 (2)	0.0504 (19)	0.0457 (18)	−0.0058 (17)	−0.0056 (16)	−0.0093 (15)
C27	0.066 (2)	0.055 (2)	0.0490 (19)	−0.0101 (18)	−0.0196 (17)	−0.0111 (16)
C28	0.054 (2)	0.062 (2)	0.060 (2)	0.0003 (18)	−0.0215 (17)	−0.0155 (17)
C29	0.059 (2)	0.0513 (19)	0.0526 (19)	−0.0038 (17)	−0.0164 (17)	−0.0110 (16)
C30	0.059 (2)	0.058 (2)	0.066 (2)	−0.0079 (18)	−0.0129 (18)	−0.0270 (17)
C31	0.054 (2)	0.068 (2)	0.074 (2)	−0.0113 (19)	−0.0163 (19)	−0.0233 (19)
C32	0.078 (3)	0.134 (4)	0.151 (4)	0.009 (3)	−0.023 (3)	−0.102 (4)

### Geometric parameters (Å, °)

**Table:**

Cl1—C14	1.736 (3)	C17—C18	1.385 (5)
Cl2—C12	1.722 (4)	C17—H17	0.9300
S3—C24	1.668 (3)	C18—C19	1.364 (5)
O4—C29	1.351 (4)	C18—H18	0.9300
O4—C32	1.436 (5)	C19—C20	1.372 (5)
N5—C13	1.398 (4)	C19—H19	0.9300
N5—C16	1.406 (4)	C20—C21	1.387 (4)
N5—H5	0.8600	C20—H20	0.9300
N6—C23	1.291 (4)	C21—C22	1.506 (5)
N6—N8	1.374 (4)	C22—C23	1.485 (4)
N7—C24	1.387 (4)	C22—H22A	0.9700
N7—C23	1.387 (4)	C22—H22B	0.9700
N7—N9	1.390 (4)	C25—C26	1.439 (5)
N8—C24	1.332 (4)	C25—H25	0.9300
N8—H8	0.8600	C26—C27	1.387 (5)
N9—C25	1.277 (4)	C26—C31	1.394 (5)
C10—C11	1.358 (6)	C27—C28	1.368 (5)
C10—C15	1.366 (6)	C27—H27	0.9300
C10—H10	0.9300	C28—C29	1.394 (5)
C11—C12	1.380 (5)	C28—H28	0.9300
C11—H11	0.9300	C29—C30	1.368 (5)
C12—C13	1.399 (4)	C30—C31	1.368 (5)
C13—C14	1.392 (4)	C30—H30	0.9300
C14—C15	1.374 (5)	C31—H31	0.9300
C15—H15	0.9300	C32—H32A	0.9600
C16—C17	1.388 (4)	C32—H32B	0.9600
C16—C21	1.405 (4)	C32—H32C	0.9600
			
C29—O4—C32	116.6 (3)	C21—C20—H20	119.0
C13—N5—C16	124.4 (3)	C20—C21—C16	118.2 (3)
C13—N5—H5	117.8	C20—C21—C22	120.7 (3)
C16—N5—H5	117.8	C16—C21—C22	121.1 (3)
C23—N6—N8	104.2 (3)	C23—C22—C21	112.3 (3)
C24—N7—C23	108.2 (3)	C23—C22—H22A	109.2
C24—N7—N9	130.0 (3)	C21—C22—H22A	109.2
C23—N7—N9	121.0 (3)	C23—C22—H22B	109.2
C24—N8—N6	114.6 (3)	C21—C22—H22B	109.2
C24—N8—H8	122.7	H22A—C22—H22B	107.9
N6—N8—H8	122.7	N6—C23—N7	110.6 (3)
C25—N9—N7	116.4 (3)	N6—C23—C22	125.3 (3)
C11—C10—C15	120.8 (4)	N7—C23—C22	123.8 (3)
C11—C10—H10	119.6	N8—C24—N7	102.3 (3)
C15—C10—H10	119.6	N8—C24—S3	129.5 (3)
C10—C11—C12	119.7 (4)	N7—C24—S3	128.2 (3)
C10—C11—H11	120.1	N9—C25—C26	122.7 (3)
C12—C11—H11	120.1	N9—C25—H25	118.6
C11—C12—C13	121.7 (4)	C26—C25—H25	118.6
C11—C12—Cl2	118.4 (3)	C27—C26—C31	117.9 (3)
C13—C12—Cl2	119.9 (3)	C27—C26—C25	123.3 (3)
C14—C13—N5	121.6 (3)	C31—C26—C25	118.8 (3)
C14—C13—C12	116.0 (3)	C28—C27—C26	120.6 (3)
N5—C13—C12	122.3 (3)	C28—C27—H27	119.7
C15—C14—C13	122.3 (3)	C26—C27—H27	119.7
C15—C14—Cl1	118.8 (3)	C27—C28—C29	120.6 (3)
C13—C14—Cl1	118.9 (3)	C27—C28—H28	119.7
C10—C15—C14	119.4 (4)	C29—C28—H28	119.7
C10—C15—H15	120.3	O4—C29—C30	124.3 (3)
C14—C15—H15	120.3	O4—C29—C28	116.3 (3)
C17—C16—C21	119.5 (3)	C30—C29—C28	119.4 (3)
C17—C16—N5	121.5 (3)	C31—C30—C29	119.9 (3)
C21—C16—N5	119.0 (3)	C31—C30—H30	120.0
C18—C17—C16	120.2 (4)	C29—C30—H30	120.0
C18—C17—H17	119.9	C30—C31—C26	121.7 (4)
C16—C17—H17	119.9	C30—C31—H31	119.2
C19—C18—C17	120.7 (4)	C26—C31—H31	119.2
C19—C18—H18	119.7	O4—C32—H32A	109.5
C17—C18—H18	119.7	O4—C32—H32B	109.5
C18—C19—C20	119.3 (3)	H32A—C32—H32B	109.5
C18—C19—H19	120.3	O4—C32—H32C	109.5
C20—C19—H19	120.3	H32A—C32—H32C	109.5
C19—C20—C21	122.1 (4)	H32B—C32—H32C	109.5
C19—C20—H20	119.0		
			
C23—N6—N8—C24	−0.3 (4)	C20—C21—C22—C23	105.1 (4)
C24—N7—N9—C25	−40.8 (4)	C16—C21—C22—C23	−77.1 (4)
C23—N7—N9—C25	150.3 (3)	N8—N6—C23—N7	−0.8 (4)
C15—C10—C11—C12	−0.2 (6)	N8—N6—C23—C22	−174.4 (3)
C10—C11—C12—C13	−1.9 (5)	C24—N7—C23—N6	1.5 (4)
C10—C11—C12—Cl2	176.8 (3)	N9—N7—C23—N6	172.7 (3)
C16—N5—C13—C14	−119.6 (3)	C24—N7—C23—C22	175.3 (3)
C16—N5—C13—C12	64.8 (4)	N9—N7—C23—C22	−13.6 (5)
C11—C12—C13—C14	2.4 (5)	C21—C22—C23—N6	87.7 (4)
Cl2—C12—C13—C14	−176.3 (2)	C21—C22—C23—N7	−85.1 (4)
C11—C12—C13—N5	178.2 (3)	N6—N8—C24—N7	1.2 (4)
Cl2—C12—C13—N5	−0.5 (4)	N6—N8—C24—S3	−177.5 (3)
N5—C13—C14—C15	−176.7 (3)	C23—N7—C24—N8	−1.6 (3)
C12—C13—C14—C15	−0.9 (5)	N9—N7—C24—N8	−171.6 (3)
N5—C13—C14—Cl1	2.2 (4)	C23—N7—C24—S3	177.1 (2)
C12—C13—C14—Cl1	178.1 (2)	N9—N7—C24—S3	7.1 (5)
C11—C10—C15—C14	1.6 (6)	N7—N9—C25—C26	179.4 (3)
C13—C14—C15—C10	−1.1 (5)	N9—C25—C26—C27	−0.4 (5)
Cl1—C14—C15—C10	180.0 (3)	N9—C25—C26—C31	179.7 (3)
C13—N5—C16—C17	7.4 (5)	C31—C26—C27—C28	−0.6 (5)
C13—N5—C16—C21	−172.8 (3)	C25—C26—C27—C28	179.5 (3)
C21—C16—C17—C18	0.2 (5)	C26—C27—C28—C29	−0.5 (5)
N5—C16—C17—C18	180.0 (3)	C32—O4—C29—C30	−1.3 (5)
C16—C17—C18—C19	−1.4 (6)	C32—O4—C29—C28	179.4 (4)
C17—C18—C19—C20	1.2 (6)	C27—C28—C29—O4	−179.7 (3)
C18—C19—C20—C21	0.2 (6)	C27—C28—C29—C30	0.9 (5)
C19—C20—C21—C16	−1.4 (5)	O4—C29—C30—C31	−179.5 (3)
C19—C20—C21—C22	176.4 (3)	C28—C29—C30—C31	−0.3 (5)
C17—C16—C21—C20	1.2 (4)	C29—C30—C31—C26	−0.8 (5)
N5—C16—C21—C20	−178.6 (3)	C27—C26—C31—C30	1.2 (5)
C17—C16—C21—C22	−176.6 (3)	C25—C26—C31—C30	−178.9 (3)
N5—C16—C21—C22	3.6 (4)		

### Hydrogen-bond geometry (Å, °)

**Table:**

Cg1 is the centroid of the triazole ring.

**Table:**

*D*—H···*A*	*D*—H	H···*A*	*D*···*A*	*D*—H···*A*
N5—H5···N6	0.86	2.35	3.047 (4)	139
N8—H8···S3^i^	0.86	2.40	3.246 (3)	170
C11—H11···Cg1^ii^	0.93	2.79	3.465 (4)	125

Symmetry codes: (i) −*x*, −*y*+2, −*z*+1; (ii) *x*+1, *y*, *z*−1.
